# Case Report: A Patient With Neuroleptic Malignant Syndrome, Water Intoxication and Hyponatremia Associated With Severe Cerebral Edema and Coma

**DOI:** 10.3389/fendo.2022.822679

**Published:** 2022-03-10

**Authors:** Haruka Takenouchi, Takatoshi Anno, Yukiko Kimura, Fumiko Kawasaki, Ryo Shirai, Hideaki Kaneto, Katsumi Kurokawa, Koichi Tomoda

**Affiliations:** ^1^ Department of General Internal Medicine 1, Kawasaki Medical School, Okayama, Japan; ^2^ Department of Diabetes, Endocrinology and Metabolism, Kawasaki Medical School, Kurashiki, Japan; ^3^ Department of Neurology, Kawasaki Medical School, Okayama, Japan

**Keywords:** severe cerebral edema, hyponatremia, neuroleptic malignant syndrome, water intoxication, syndrome of inappropriate secretion of antidiuretic hormone

## Abstract

**Background:**

Water intoxication is typically caused by primary or psychogenic polydipsia that potentially may lead to fatal disturbance in brain functions. Neuroleptic malignant syndrome (NMS) is a serious complication induced by administration of antipsychotics and other psychotropic drugs. The combination of inappropriate secretion of antidiuretic hormone (SIDAH), NMS and rhabdomyolysis have been rarely reported. Our patient also developed severe water intoxication.

**Case presentation:**

Herein we report a comatose case of NMS complicated with water intoxication, syndrome of SIADH and rhabdomyolysis. This patient had severe cerebral edema and hyponatremia that were improved rapidly by the correction of hyponatremia within a couple of days.

**Conclusions:**

Malignant neuroleptic syndrome water intoxication, SIADH and rhabdomyolysis can occur simultaneously. Comatose conditions induced by cerebral edema and hyponatremia can be successfully treated by meticulous fluid management and the correction of hyponatremia.

## Background

Water intoxication is a condition that is typically caused by primary or psychogenic polydipsia. In addition, water intoxication potentially disturbs brain function when the abnormal balance of electrolytes, especially hyponatremia and hypo osmotic pressure, is brought about by excessive water intake (1). It is known that water intoxication occurs in 15–25% of patients with chronic mental diseases as a result of compulsive drinking (2). Neuroleptic malignant syndrome (NMS) is a serious complication induced by administration of antipsychotics and other psychotropic drugs (3). Symptoms of NMS include myotonicity, hyperthermia, altered consciousness, and autonomic nervous system symptoms. NMS is also complicated with hyponatremia and water intoxication (4, 5). The combination of syndrome of inappropriate secretion of antidiuretic hormone (SIADH), NMS and rhabdomyolysis have been rarely reported (6).

Herein we report a case of NMS complicated with water intoxication, cerebral edema and hyponatremia. His coma and severe cerebral edema were dramatically improved by the correction of hyponatremia within 3 days.

## Case Presentation

**Figure 1:**
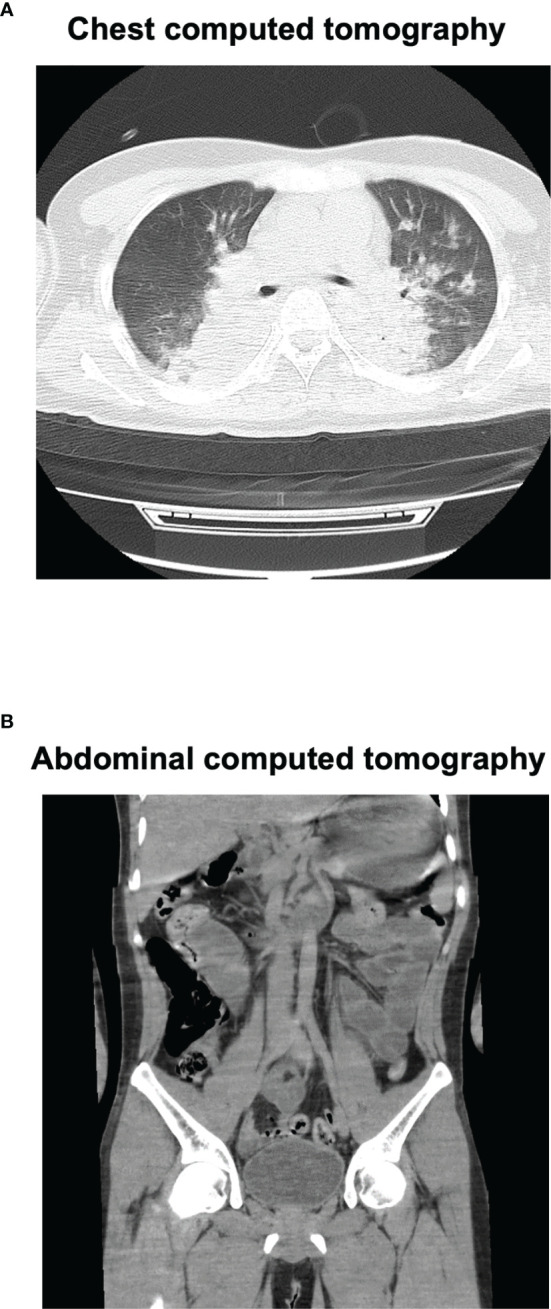
Chest and abdominal computed tomography (CT) on admission. Chest CT **(A)** revealed marked pleural effusion and pulmonary edema. Abdominal CT **(B)** revealed marked intestinal edema.

A 27-year-old Japanese man was brought to the emergency room with coma. He was diagnosed as having schizophrenia at the age of 13 and as having autism spectrum disorder at the age of 18. The medication at that time was 50 mg/day of atomoxetine, 5 mg/day of risperidone and 3 mg/day of guanfacine, although risperidone was decreased from 9 to 5 mg and guanfacine was increased from 2 to 3 mg 35 days before. He had been drinking over 4 L of water for the last few weeks. His impaired consciousness level was 3 points (E1V1M1) of the Glasgow coma scale (GCS). His vital signs were as follows: temperature, 39.1°C; blood pressure, 174/98 mmHg; heart rate, 95 beats/min; oxygen saturation, 92% (under 9 L of O_2_ supply). **Table 1** shows laboratory data in emergency room. He suffered from abnormal balance of electrolytes. As shown in **Table 1**, he had significant hyponatremia and hypochloremia with normal potassium concentration, and reduction of blood osmotic pressure. His inflammation markers were markedly elevated. In addition, he suffered from rhabdomyolysis. Indeed, his creatine kinase, myoglobin and urinary myoglobin levels were significantly high. As shown in **Figure 1**, his head computed tomography (CT) (**Figure 1A**) revealed severe cerebral edema. In addition, significant fluid retention was observed throughout the body, namely, pleural effusion, pulmonary and intestinal edema (**Figures 1A, B**). We evaluated the causes of hyponatremia in this patient. Since antidiuretic hormone (ADH) level was elevated to 0.8 pg/ml when sodium level was 109 mmol/L, we diagnosed him with syndrome of inappropriate secretion of ADH (SIADH). Based on these findings, we thought that he probably suffered from severe cerebral edema which was induced by hyponatremia associated with NMS and SIADH. In addition, rhabdomyolysis and NMS was diagnosed necessitating comprehensive therapy in intensive care unit (ICU).

**Table 1:** Laboratory data in an emergency room in this subject.

Variable	Result	Reference range	Variable	Result	Reference range
**Peripheral blood**			**Blood biochemistry**		
White blood cells (μl)	14,510	3,300–8,600	Plasma glucose (mg/dl)	107	
Neutrophil (%)	92.0	52.0–80.0	Total cholesterol (mg/dl)	122	142–248
Red blood cells (×10^4^/μl)	509	435–555	LDL cholesterol (mg/dl)	45	65–139
Hemoglobin (g/dl)	15.7	13.7–16.8	HDL cholesterol (mg/dl)	62	40–90
Hematocrit (%)	40.3	40.7–50.1	Triglyceride (mg/dl)	33	40–149
Platelets (×10^4^/μl)	22.8	15.8–34.8	CRP (mg/dl)	7.69	<0.14
**Blood biochemistry**			Procalcitonin (ng/ml)	0.12	0.00–0.05
Total protein (g/dl)	7.0	6.6–8.1	**Blood Gas Analysis**		
Albumin (g/dl)	4.5	4.1–5.1	pH	7.382	7.360–7.460
Globulin (g/dl)	2.5	2.2–3.4	PCO_2_ (mmHg)	29.7	34.0–46.0
Total bilirubin (mg/dl)	1.4	0.4–1.5	PO_2_ (mmHg)	54.1	80.0–90.0
Direct bilirubin (%)	9	30–52	HCO_3_^−^ (mEq/L)	17.2	24.0–32.0
AST (U/L)	311	13–30	BE (mEq/L)	−6.1	−2.5–2.5
ALT (U/L)	50	10–42	SO_2_ (%)	85.1	95.0–98.0
LDH (U/L)	784	124–222	Lactate (mEq/L)	2.30	0.63–2.44
ALP (U/L)	481	106–322	**Coagulation fibrinolytic system**		
γ-GTP (U/L)	12	13–64	PT-sec (s)	14.1	9.3–12.5
BUN (mg/dl)	5	8–20	PT-INR	1.27	0.85–1.13
Creatinine (mg/dl)	0.57	0.65–1.07	PT-activity (%)	65.6	80.7–125.2
Cholinesterase (U/L)	299	240–486	APTT (sec)	46.9	26.9–38.1
Uric acid (mg/dl)	6.3	3.7–7.8	Fibrinogen (mg/dl)	281	160–380
Creatine Kinase (U/L)	26,110	59–248	D-dimer (μg/ml)	2.30	<1.0
Amylase (μg/dl)	135	44–132	**Urinary test**		
P-amylase (IU/L37°C)	21	19–53	Urinary pH	6.0	5.0–7.5
Ammonia (μg/dl)	68	12–66	Urinary protein	1+	–
Sodium (mmol/L)	109	138–145	Urinary sugar	–	–
Potassium (mmol/L)	4.5	3.6–4.8	Urinary ketone body	2+	–
Chloride (mmol/L)	81	101–108	Urinary bilirubin	–	–
Calcium (mg/dl)	8.0	8.8–10.1	Urinary blood	3+	–
S-osmolality (mOsm/kg)	228	277–295	U-osmolality (mOsm/kg)	627	
S-myoglobin (ng/ml)	24,040	0.0–154.9	U-myoglobin (μg/L)	920,000	0–200

AST, aspartate aminotransferase; ALT, alanine aminotransferase; LDH, lactate dehydrogenase; ALP, alkaline phosphatase; γ-GTP, γ-glutamyl transpeptidase; BUN, blood urea nitrogen; P- amylase, Pancreatic amylase; S-osmolality, Serum osmolality; S-myoglobin, Serum myoglobin; LDL, Low-density lipoprotein; HDL, High-density lipoprotein; CRP, C-reactive protein; BE, Base Excess; PT, prothrombin; PT-INR, PT-international normalized ratio; APTT, activated partial thromboplastin time; U-osmolality, Urinary osmolality; U-myoglobin, Urinary myoglobin.

**Figure 2:**
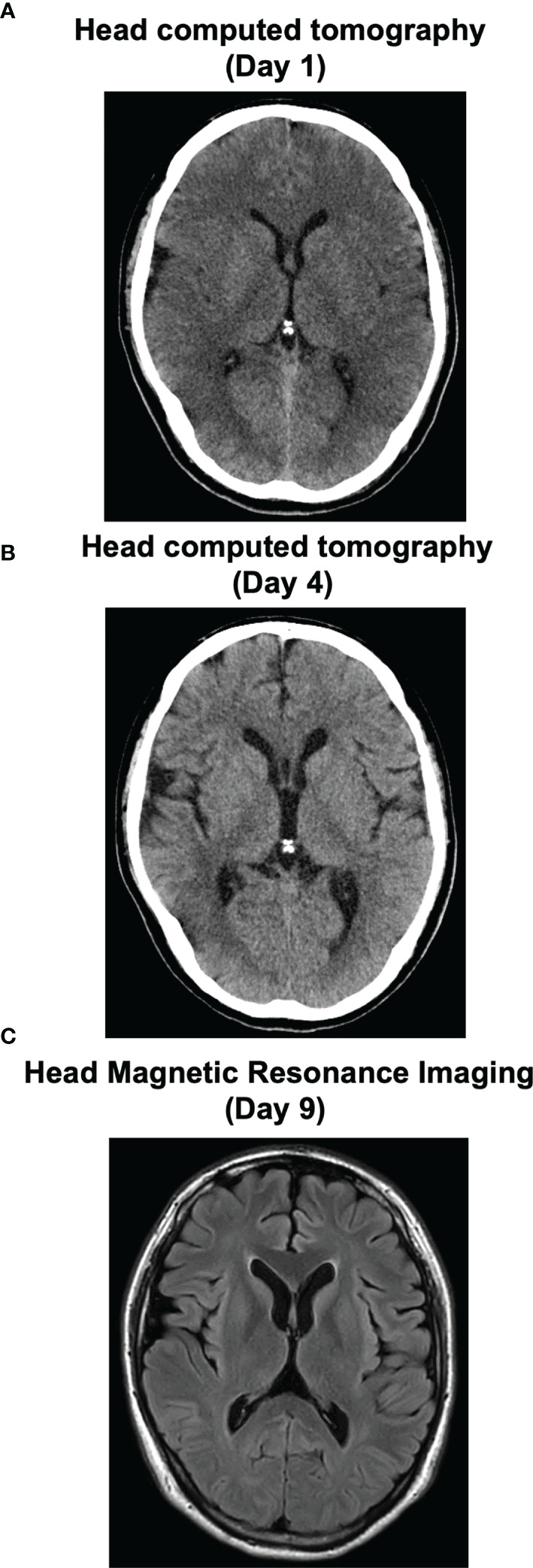
Time course of cerebral edema in this subject on the image inspection. His severe cerebral edema on admission **(A)** was improved at day 4 **(B)**. At day 9, he was transferred from HCU to general ward. His head magnetic resonance imaging was normal **(C)**.

**Figure 3:**
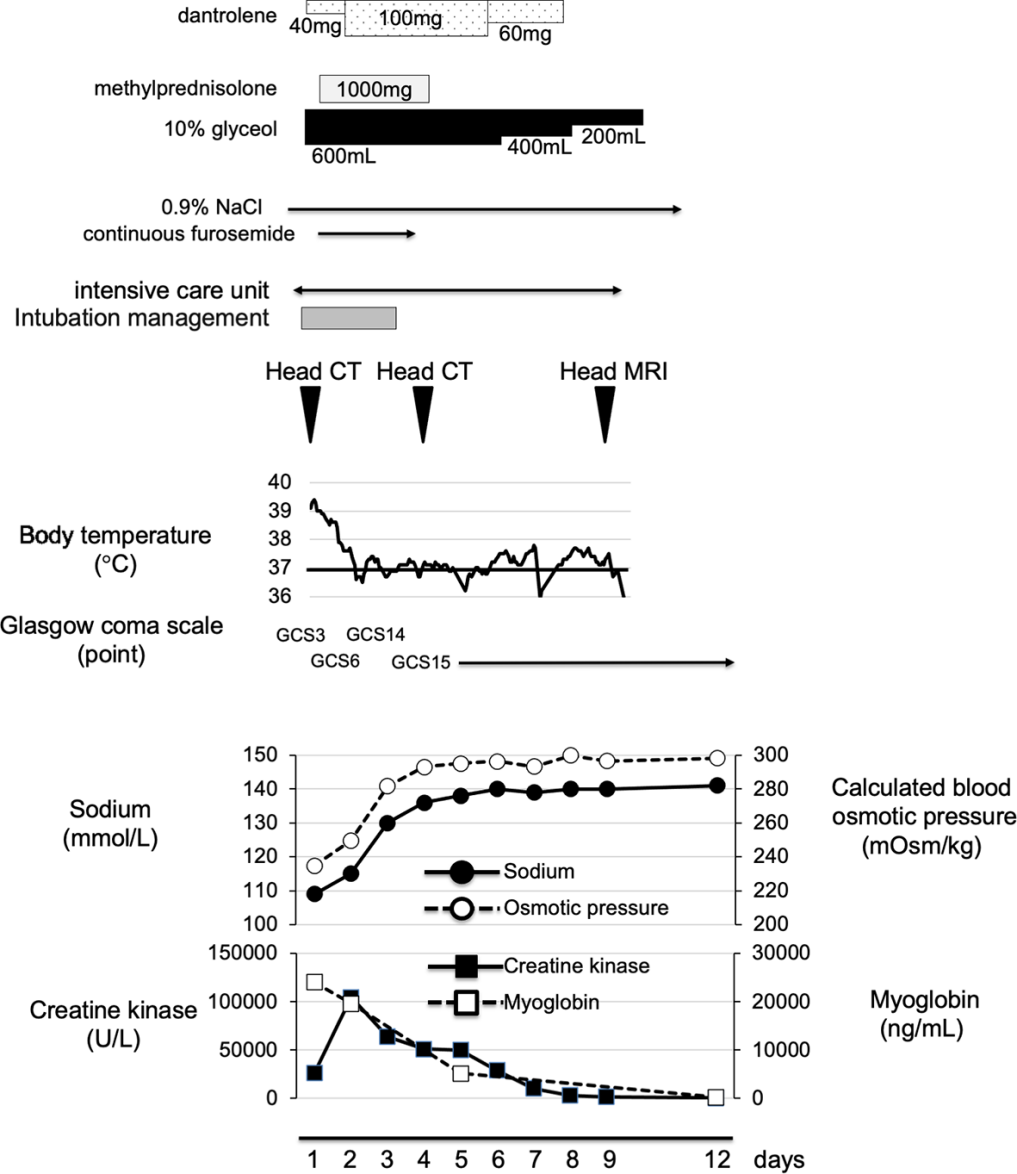
Time course of clinical parameters in this subject. On admission, we started fluid management, by using 10% glyceol and methylprednisolone for cerebral edema and dantrolene for NMS. His body temperature and coma were improved together with correction of hyponatremia and cerebral edema. He was transferred from HCU to general ward at day 9.

On admission to ICU, he required mechanical ventilation and administration of 0.9% NaCl. Moreover, we started immediately administering 10% glyceol (600 ml/once a day, 5 days + 400 ml/once a day, 2 days + 200 ml/once a day, 2 days) for cerebral edema and continuous furosemide (3 days) for diuresis. In addition, he was treated with methylprednisolone (1,000 mg/once a day, 3 days) for cerebral edema and suspection of autoimmune encephalitis, and with dantrolene (40 mg/once a day, 1 day + 100 mg/once a day, 4 days + 60 mg/once a day, 2 days) for NMS. Eye openings and spontaneous limb movements were observed several times at day 2 (his sodium level, 115 mmol/L). His impaired consciousness was improved and he was able to speak sometimes at day 3 (his sodium level, 130 mmol/L). The patient was extubated at day 3 and his head CT revealed the improvement of severe cerebral edema at day 4 (**Figure 2B**). Finally, we successfully treated severe cerebral edema and hyponatremia, which was induced and complicated with NMS, water intoxication, SIADH and rhabdomyolysis. **Figure 2** shows a time course of his cerebral edema and **Figure 3** shows his clinical time course in ICU. His adrenal and thyroid function was normal (adrenocorticotropic hormone, 61.7 pg/ml; cortisol, 13.0 μg/dl; thyroid stimulating hormone, 3.208 μIU/ml; free triiodothyronine, 2.89 pg/ml; free thyroxine, 1.03 ng/dl; respectively) after correction of hyponatremia. He was transferred from ICU to general ward at day 9 and was discharged 29 days after admission.

After discharge, he was followed-up by the psychosomatic center of another hospital. He continued to receive the same psychosomatic treatment and did not have recurrence of malignant syndromes or water intoxication.

## Discussion

NMS is a serious complication that is estimated to occur in 0.07 to 2.2% of patients mainly after administration of antipsychotics and other psychotropic drugs (7, 8). Although the pathogenesis and the degree of progression are various, the prognosis of severe NMS is poor when water intoxication and rhabdomyolysis are complicated. On the other hand, water intoxication occurs in about 20% of patients with mental illness who are hospitalized for a long period of time (9), and in about 15% of outpatients with such disorders (10). Water intoxication is often complicated with hyponatremia and hypo osmotic pressure, and is reported to be associated with SIADH (11). There are few case reports showing complication of NMS and water intoxication (5, 12–17). Moreover, rhabdomyolysis is sometimes complicated together with both NMS and water intoxication (12, 14, 18). Our patient was a rare case in that he had NMS, water intoxication (namely, hyponatremia and hypo osmotic pressure), SIADH and rhabdomyolysis at the same time.

Most severe condition of our patient was impaired consciousness [3 points (E1V1M1) of GCS] and severe cerebral edema. In this point, we considered severe cerebral edema was mainly caused by hyponatremia due to water intoxication and SIADH. Therefore, we mainly performed fluid management by gradually improving abnormal balance of electrolytes and hyponatremia and reduced cerebral edema by using glyceol, methylprednisolone and furosemide. Moreover, since NMS was also considered as a possible cause of impaired consciousness, we started administering dantrolene. Eye openings and spontaneous limb movements [GCS6(E3VTM3)] were observed several times in the next day. The presence of rhabdomyolysis also made treatment difficult, and the first choice of treatment for rhabdomyolysis was aggressive hydration, although we would like to refrain from hydrating against cerebral edema and SIADH. Three days later, his impaired consciousness was improved and we performed his extubation [GCS14(E4V4M6)]. We performed head CT 4 days after admission, and we made sure that we successfully treated severe cerebral edema with correction of hyponatremia. In cerebral edema caused by hyponatremia, there is a risk of developing cerebral herniation due to exacerbation of cerebral edema and osmotic demyelination syndrome (ODS) during the treatment process. Once ODS is developed, about half of the patients may die or have residual sequelae. Therefore, we should correct hyponatremia slowly (19). We were able to successfully improve severe cerebral edema with fluid management focusing on the correction of hyponatremia. More interestingly, his impaired consciousness was improved dramatically in a short period of time as the cerebral edema improved.

There is a limitation in this case report. We examined adrenal and thyroid function after hyponatremia was corrected. In the pathogenesis of NMS, hypothyroidism and adrenal insufficiency have also been implicated (20). Since we did not check adrenal and thyroid function before starting methylprednisolone, we failed to completely exclude the possibility of hypothyroidism and adrenal insufficiency. However, since his adrenal and thyroid function was normal although they were evaluated after starting methylprednisolone, we thought that hypothyroidism or adrenal insufficiency was less likely to associated with NMS. Moreover, it is known that patients with acute psychosis have a propensity to develop hyponatremia (21), and some of psychogenic drugs can cause hyponatremia by unknown mechanism (22). In the pathogenesis of NMS, preceding hyponatremia could be a trigger for NMS (18).

Taken together, we should bear in mind that NMS, water intoxication, SIADH, and rhabdomyolysis can occur at the same time. In such a case, severe cerebral edema induced by hyponatremia and NMS may be the cause of coma. Furthermore, although slow correction of hyponatremia is necessary, fluid management, especially correction of hyponatremia, may dramatically improve cerebral edema and coma. Therefore, it is important to perform early diagnosis of cerebral edema due to hyponatremia, and it is necessary to be prepared for all conditions of whole-body management.

## Data Availability Statement

The original contributions presented in the study are included in the article/supplementary material. Further inquiries can be directed to the corresponding author.

## Ethics Statement

Written informed consent was obtained from the individual(s) for the publication of any potentially identifiable images or data included in this article.

## Author Contributions

HT and TA researched data and wrote the manuscript. YK, FK, RS, and KK researched data and contributed to the discussion. HK, KK, and KT reviewed the manuscript. All authors listed have made a substantial, direct, and intellectual contribution to the work and approved it for publication.

## Conflict of Interest

The authors declare that the research was conducted in the absence of any commercial or financial relationships that could be construed as a potential conflict of interest.

## Publisher’s Note

All claims expressed in this article are solely those of the authors and do not necessarily represent those of their affiliated organizations, or those of the publisher, the editors and the reviewers. Any product that may be evaluated in this article, or claim that may be made by its manufacturer, is not guaranteed or endorsed by the publisher.
